# Draft genome sequences of a historical collection of *Listeria monocytogenes* from humans and other sources, 1926–1964

**DOI:** 10.1128/MRA.00625-23

**Published:** 2023-09-29

**Authors:** Phillip Brown, Robert G. E. Murray, Sara Galsworthy, Mirena Ivanova, Pimlapas Leekitcharoenphon, Todd Ward, Zuzana Kucerova, Yi Chen, Driss Elhanafi, Robin Siletzky, Sophia Kathariou

**Affiliations:** 1 Department of Plant and Microbial Biology, North Carolina State University, Raleigh, North Carolina, USA; 2 Department of Microbiology and Immunology, University of Western Ontario, London, Ontario, Canada; 3 Research Group for Genomic Epidemiology, National Food Institute,Technical University of Denmark, Lyngby, Denmark; 4 U.S. Department of Agriculture, Agricultural Research Service, Peoria, Illinois, USA; 5 Centers for Disease Control and Prevention (CDC), EDLB/DFWED, Atlanta, Georgia, USA; 6 Division of Microbiology, Center for Food Safety and Applied Nutrition, Food and Drug Administration, College Park, Maryland, USA; 7 Biomanufacturing Training and Education Center, North Carolina State University, Raleigh, North Carolina, USA; 8 Department of Food, Bioprocessing and Nutrition Sciences, North Carolina State University, Raleigh, North Carolina, USA; Queens College, Queens, New York, USA

**Keywords:** *Listeria monocytogenes*, food-borne pathogens, listeriosis, DNA sequencing, genomes, intracellular pathogens, historical strains

## Abstract

*Listeria monocytogenes* can persistently contaminate food processing environments and tolerate sanitizers. Most sequenced strains are from clinical and environmental sources in the contemporary era, with relatively few prior to extensive food processing and sanitizer use. We report the genome sequences of a diverse panel of 83 strains from 1926 to 1964.

## ANNOUNCEMENT

*Listeria monocytogenes* is a foodborne facultative intracellular bacterial pathogen responsible for severe disease (listeriosis), with symptoms that include meningitis, septicemia, and stillbirths. Pregnancy, advanced age, and immunosuppression are major risk factors for listeriosis (1, 2). *L. monocytogenes* is notorious for its persistence in food processing environments (FPEs) via various adaptations including resistance to quaternary ammonium compounds (QACs) (3). The first outbreak of human listeriosis to confirm foodborne transmission of *L. monocytogenes* was in 1981 in the Maritime Provinces, Canada (4). Most sequenced strains were from subsequent periods, characterized by increasing QAC use in FPEs, with relatively few prior to extensive food processing and sanitizer use (5, 6). Inclusion of such strains is valuable to the analysis of disease agents (7). Here, we report the genome sequences of a historical collection of 83 *L*. *monocytogenes* strains from human listeriosis and other sources, 1926–1964 (Table 1). Two additional strains from the same collection were reported previously (8). Almost half of the strains belonged to the hypervirulent clonal complexes CC1 (*n* = 22) and CC2 (*n* = 15) (Table 1). CC1 and CC2 strains were from various sources (human clinical, animals, environment) and regions (North America, Europe, Asia), over three decades (1933–1964), in agreement with these being ancient, widely distributed clones (9). Interestingly, 12 novel sequence types (STs) were identified, even though these strains being isolated over 50 years ago. Numerous strains, primarily CC1 and CC2, were resistant to cadmium and arsenic, while none exhibited QAC resistance (Table 1).

Strains were resuscitated from the lyophilized laboratory collection of R.G.E. Murray by streaking a portion of the lyophilizate on tryptic soy (TS) (Becton, Dickinson & Co., Sparks, MD, USA) agar with 4% sheep blood agar and incubation at 37°C for 36–48 h. For sequencing, frozen culture stocks (−80°C) were streaked on TS agar and incubated at 37°C overnight. Genomic DNA was extracted by the DNeasy blood and tissue kit (Qiagen, Valencia, CA, USA) from strains grown overnight at 37°C in TS or brain heart infusion (Becton, Dickinson & Co.) broth according to the manufacturer’s instructions. Libraries were prepared using 0.5–1 ng of genomic DNA with a Nextera XT DNA library preparation kit (Illumina, San Diego, CA, USA). Genomes were sequenced using a NextSeq 500 sequencer with the NextSeq 500/550 high-output kit v2.5 (300 cycles, 2 × 150 bp) (Illumina) or a MiSeq desktop sequencer with the MiSeq kit v2 (500 cycles, 2 × 250 bp) (Illumina) according to the manufacturer’s instructions (Table 1). Raw sequencing reads were quality controlled by FastQC v0.11.5 (10) and trimmed using bbduk2 from bbtools v38.89 (sourceforge.net/projects/bbmap/), CLC Genomics Workbench 7.5.1 (CLC Bio, Boston, MA, USA) or BioNumerics 7.6.3 (BioMérieux, Sint-Martens-Latem, Belgium) (Table 1). Reads were deposited into the National Center for Biotechnology Information (https://www.ncbi.nlm.nih.gov), assembled by SKESA v2.2 (11), and annotated using the NCBI Prokaryotic Genome Annotation Pipeline v6.3 (12). ST and CC designations were determined using BIGSdb-Lm, hosted by Institut Pasteur (https://bigsdb.pasteur.fr/listeria/) (13). Default parameters were used for all software. Resistance to cadmium and arsenic was determined as described (14).

**TABLE 1:** Characteristics of the 83 historical *Listeria monocytogenes* strains described in this report

Strain	ST^*a*^	CC^*b*^	Serotype^*c*^	Year	Source	Cd35/70^*d*^	BC10^*d*^	As500^*d*^	No. of contigs	Total length (bp)	GC content (%)	N50 (bp)	Coverage (x)	No. of reads	Sequencing method	Read quality control and trimming tools	GenBank accession	BioSample accession	Assembly accession	Sequence read archive accession
OLM38	7	7	1/2a	1938	Calf, Iowa, USA	−	−	−	47	2,956,055	37.9	168,662	90	912,493	NextSeq 500	FastQC v0.11.5, bbduk v38.89	DAKLVL000000000	SAMN31902370	GCA_026550595.1	SRR22438322
OLM55	7	7	1/2a	1951	Human clinical	+	−	−	44	3,036,917	37.8	163,896	113	1,199,851	NextSeq 500	FastQC v0.11.5, bbduk v38.89	DAKLVC000000000	SAMN31902380	GCA_026550375.1	SRR22438330
OLM70	7	7	1/2a	1953	Cow fetus, Israel	−	−	−	42	2,901,112	37.9	138,323	94	932,129	NextSeq 500	FastQC v0.11.5, bbduk v38.89	DAKLVO000000000	SAMN31902390	GCA_026550735.1	SRR22438342
OLM75	7	7	1/2a	1952	Human clinical, Montreal, CA	−	−	−	16	3,004,781	37.9	436,919	224	2,407,104	NextSeq 500	CLC Genomics Workbench 7.5.1	ABJNTC000000000	SAMN31821595	GCA_026379275.1	SRR22362544
OLM81	98	7	1/2a	1954	Unknown	−	−	−	96	2,889,243	37.9	58,658	122	1,208,524	NextSeq 500	FastQC v0.11.5, bbduk v38.89	DAKLUM000000000	SAMN31902399	GCA_026550095.1	SRR22438351
OLM3	158	7	1/2a	1926	Gerbil, Tiger River District, South Africa	−	−	−	33	2,907,513	37.9	342,894	108	1,068,854	NextSeq 500	FastQC v0.11.5, bbduk v38.89	DAKLUD000000000	SAMN31902412	GCA_026549935.1	SRR22438360
OLM137	35	9	1/2a	1960	Cow, Nova Scotia, CA	+	−	−	58	2,976,736	37.8	86,045	113	1,143,940	NextSeq 500	FastQC v0.11.5, bbduk v38.89	DAKLVZ000000000	SAMN31902350	GCA_026550915.1	SRR22438310
OLM25	122	9	1/2a	1938	Human clinical, Scotland	+	−	−	123	2,997,108	37.8	41,286	86	880,711	NextSeq 500	FastQC v0.11.5, bbduk v38.89	DAKLWO000000000	SAMN31902362	GCA_026551175.1	SRR22438297
OLM67	91	14	1/2a	1953	Chinchilla, Ontario, CA	−	−	−	37	2,888,088	37.9	175,223	104	1,028,154	NextSeq 500	FastQC v0.11.5, bbduk v38.89	DAKLUU000000000	SAMN31902388	GCA_026550215.1	SRR22438344
OLM72	91	14	1/2a	1937	Rat, Ontario, CA	−	−	−	33	2,877,877	37.9	245,684	119	1,207,962	NextSeq 500	FastQC v0.11.5, bbduk v38.89	DAKLUR000000000	SAMN31902392	GCA_026550255.1	SRR22438340
OLM73	91	14	1/2a	1953	Fowl, Alberta, CA	−	−	−	47	2,955,446	37.9	107,607	119	1,214,757	NextSeq 500	FastQC v0.11.5, bbduk v38.89	DAKLUW000000000	SAMN31902393	GCA_026550295.1	SRR22438339
OLM26	545	14	1/2a	1938	Hen, England	−	−	−	80	2,928,483	37.9	63,825	95	956,591	NextSeq 500	FastQC v0.11.5, bbduk v38.89	DAKLWQ000000000	SAMN31902363	GCA_026551195.1	SRR22438296
OLM46	**2969**	14	1/2a	1950	Lemming, Arctic, CA	−	−	−	74	2,976,429	38.0	70,963	101	1,026,437	NextSeq 500	FastQC v0.11.5, bbduk v38.89	DAKLVA000000000	SAMN31902376	GCA_026550435.1	SRR22438334
OLM28	325	31	1/2a	1938	Pullet, England	+	−	−	70	3,018,586	37.8	83,462	77	794,275	NextSeq 500	FastQC v0.11.5, bbduk v38.89	DAKLWP000000000	SAMN31902364	GCA_026551215.1	SRR22438295
OLM29	325	31	1/2a	1938	Pullet, England	+	−	+	61	3,006,883	37.8	104,941	113	1,169,913	NextSeq 500	FastQC v0.11.5, bbduk v38.89	DAKLWR000000000	SAMN31902365	GCA_026551235.1	SRR22438294
OLM87	101	101	1/2a	1954	Human clinical, CT, USA	−	−	−	56	2,937,984	37.8	120,699	129	1,326,321	NextSeq 500	FastQC v0.11.5, bbduk v38.89	DAKLUP000000000	SAMN31902402	GCA_026550175.1	SRR22438348
OLM52d	103	101	1/2a	1951	Clinical, Halle, Germany	−	−	−	147	2,915,597	37.9	36,539	56	571,706	NextSeq 500	FastQC v0.11.5, bbduk v38.89	DAKLVD000000000	SAMN31902378	GCA_026550395.1	SRR22438332
OLM54d	103	101	1/2a	1951	Human clinical	−	−	−	62	2,941,902	37.9	86,970	81	813,938	NextSeq 500	FastQC v0.11.5, bbduk v38.89	DAKLVE000000000	SAMN31902379	GCA_026550355.1	SRR22438331
OLM56	**2996**	101	1/2a	1951	Human clinical	−	−	−	54	2,936,283	37.9	108,804	101	1,024,080	NextSeq 500	FastQC v0.11.5, bbduk v38.89	DAKLVG000000000	SAMN31902381	GCA_026550415.1	SRR22438329
OLM42	155	155	1/2a	1948	Human clinical, Australia	ND	ND	ND	65	3,001,219	37.8	114,668	99	1,009,565	NextSeq 500	FastQC v0.11.5, bbduk v38.89	DAKLVM000000000	SAMN31902373	GCA_026550655.1	SRR22438319
OLM51	155	155	1/2a	1948	Human clinical, Canada	−	−	−	59	2,960,167	37.8	132,886	101	1,025,421	NextSeq 500	FastQC v0.11.5, bbduk v38.89	DAKLUZ000000000	SAMN31902377	GCA_026550315.1	SRR22438333
OLM128	59	59	1/2b	1959	Moose, Nova Scotia, CA	+	−	−	75	2,992,838	37.9	75,507	123	1,265,841	NextSeq 500	FastQC v0.11.5, bbduk v38.89	DAKLXW000000000	SAMN31902347	GCA_026552055.1	SRR22438313
OLM135	**2997**	59	1/2b	1955	Human clinical, MI, USA	+	−	−	38	2,921,018	37.9	183,046	106	1,057,297	NextSeq 500	FastQC v0.11.5, bbduk v38.89	DAKLWB000000000	SAMN31902348	GCA_026550855.1	SRR22438312
OLM136	**2966**	344	1/2b	1960	Human clinical, LA, USA	−	−	−	67	2,981,247	37.8	90,970	95	971,643	NextSeq 500	FastQC v0.11.5, bbduk v38.89	DAKLWA000000000	SAMN31902349	GCA_026550895.1	SRR22438311
OLM31	9	9	1/2c	1939	Human clinical, Scotland	−	−	−	41	2,902,409	37.9	158,004	98	972,990	NextSeq 500	FastQC v0.11.5, bbduk v38.89	DAKLVB000000000	SAMN31902367	GCA_026550495.1	SRR22438335
OLM41	9	9	1/2c	1949	Canary, Alberta, CA	ND	ND	ND	25	2,978,479	37.9	344,017	69	745,042	NextSeq 500	FastQC v0.11.5, bbduk v38.89	DAKLVP000000000	SAMN31902372	GCA_026550615.1	SRR22438320
OLM76	9	9	1/2c	1952	Human clinical	+	−	−	62	3,068,075	37.9	112,971	80	838,936	NextSeq 500	FastQC v0.11.5, bbduk v38.89	DAKLUX000000000	SAMN31902395	GCA_026550455.1	SRR22438337
OLM13	478	9	1/2c	1933	Human clinical, USA	−	−	−	37	2,940,656	37.9	188,694	106	1,060,750	NextSeq 500	FastQC v0.11.5, bbduk v38.89	DAKLTY000000000	SAMN31902407	GCA_026549775.1	SRR22438366
OLM125	1	1	4b	1959	Clinical, infant, Ontario, CA	+	−	+	47	2,975,346	37.9	134,804	96	974,032	NextSeq 500	FastQC v0.11.5, bbduk v38.89	DAKLWW000000000	SAMN31902344	GCA_026551335.1	SRR22438288
OLM126	1	1	4b	1959	Sheep, Nova Scotia, CA	−	−	−	42	2,920,082	37.9	167,290	104	1,037,749	NextSeq 500	FastQC v0.11.5, bbduk v38.89	DAKLWY000000000	SAMN31902345	GCA_026551375.1	SRR22438287
OLM141	1	1	4b	1956	Mud, Bucharest, Romania	−	−	−	98	2,985,047	37.9	62,684	148	1,544,143	NextSeq 500	FastQC v0.11.5, bbduk v38.89	DAKLWC000000000	SAMN31902352	GCA_026550935.1	SRR22438308
OLM142	1	1	4b	1960	Human clinical, Newfoundland, CA	+	−	+	114	2,954,876	37.9	58,336	105	1,055,177	NextSeq 500	FastQC v0.11.5, bbduk v38.89	DAKLWI000000000	SAMN31902353	GCA_026550975.1	SRR22438307
OLM143	1	1	4b	1961	Human clinical, Ontario, CA	−	−	−	115	2,926,938	37.9	52,946	93	931,130	NextSeq 500	FastQC v0.11.5, bbduk v38.89	DAKLWE000000000	SAMN31902354	GCA_026550995.1	SRR22438306
OLM147	1	1	4b	1961	Human clinical, BC, CA	+	−	+	76	2,960,107	37.9	73,603	93	936,256	NextSeq 500	FastQC v0.11.5, bbduk v38.89	DAKLWF000000000	SAMN31902356	GCA_026551055.1	SRR22438303
OLM152	1	1	4b	1963	Human clinical, Newfoundland, CA	+	−	+	106	2,970,595	37.9	57,633	106	1,145,820	NextSeq 500	FastQC v0.11.5, bbduk v38.89	DAKLWN000000000	SAMN31902358	GCA_026551095.1	SRR22438301
OLM43	1	1	4b	1949	Cow, Ontario, CA	−	−	−	86	2,888,414	37.9	69,000	85	835,289	NextSeq 500	FastQC v0.11.5, bbduk v38.89	DAKLVW000000000	SAMN31902374	GCA_026550755.1	SRR22438318
OLM61	1	1	4b	1951	Human clinical, Ontario, CA	−	−	+	68	2,988,342	37.9	76,106	97	986,907	NextSeq 500	FastQC v0.11.5, bbduk v38.89	DAKLVR000000000	SAMN31902383	GCA_026550795.1	SRR22438327
OLM66	1	1	4b	1953	Sheep, Japan	−	−	−	133	2,918,869	37.9	45,980	74	737,302	NextSeq 500	FastQC v0.11.5, bbduk v38.89	DAKLVQ000000000	SAMN31902387	GCA_026550695.1	SRR22438355
OLM69	1	1	4b	1952	Cow fetus, Israel	−	−	−	50	2,924,367	37.9	121,659	120	1,212,792	NextSeq 500	FastQC v0.11.5, bbduk v38.89	DAKLUS000000000	SAMN31902389	GCA_026550155.1	SRR22438343
OLM71	1	1	4b	1953	Clinical, infant, Ontario, CA	+	−	−	64	3,056,320	37.8	96,191	83	866,139	NextSeq 500	FastQC v0.11.5, bbduk v38.89	DAKLUV000000000	SAMN31902391	GCA_026550235.1	SRR22438341
OLM74	1	1	4b	1953	Human clinical, Ontario, CA	+	−	−	52	3,032,681	37.9	145,284	86	894,094	NextSeq 500	FastQC v0.11.5, bbduk v38.89	DAKLUT000000000	SAMN31902394	GCA_026550275.1	SRR22438338
OLM97	1	1	4b	1954	Human clinical, Nova Scotia, CA	+	−	−	23	2,956,449	37.9	238,200	92	927,198	NextSeq 500	FastQC v0.11.5, bbduk v38.89	DAKLVN000000000	SAMN31902404	GCA_026550675.1	SRR22438346
OLM98	1	1	4b	1955	Human clinical, Nova Scotia, CA	+	−	+	55	2,967,740	37.9	122,829	92	934,499	NextSeq 500	FastQC v0.11.5, bbduk v38.89	DAKLVS000000000	SAMN31902405	GCA_026550715.1	SRR22438345
OLM63	63	1	4b	1951	Cow, Japan	−	−	−	39	2,894,693	37.9	159,583	77	754,512	NextSeq 500	FastQC v0.11.5, bbduk v38.89	DAKLVI000000000	SAMN31902385	GCA_026550535.1	SRR22438325
OLM9	73	1	4b	1933	Calf, USA	+	−	+	57	2,948,419	37.9	107,737	79	788,565	NextSeq 500	FastQC v0.11.5, bbduk v38.89	DAKLTZ000000000	SAMN31902416	GCA_026549835.1	SRR22438365
OLM20	278	1	4b	1937	Sheep, USA	−	−	−	35	2,915,948	37.9	178,553	101	997,240	NextSeq 500	FastQC v0.11.5, bbduk v38.89	DAKLUF000000000	SAMN31902410	GCA_026549915.1	SRR22438362
OLM15	308	1	4b	1934	Clinical, infant, USA	+	−	+	76	2,980,222	37.9	83,714	65	655,768	NextSeq 500	FastQC v0.11.5, bbduk v38.89	DAKLUA000000000	SAMN31902408	GCA_026549855.1	SRR22438364
OLM18	308	1	4b	1934	Clinical, infant, USA	+	−	−	47	3,009,556	37.8	140,846	90	923,262	NextSeq 500	FastQC v0.11.5, bbduk v38.89	DAKLUE000000000	SAMN31902409	GCA_026549875.1	SRR22438363
OLM93	308	1	4b	1954	Human clinical, Ontario, CA	−	−	+	84	2,941,466	37.9	65,257	137	1,401,328	NextSeq 500	FastQC v0.11.5, bbduk v38.89	DAKLUQ000000000	SAMN31902403	GCA_026550195.1	SRR22438347
OLM65	**2099**	1	4b	1952	Goat, Japan	−	−	−	34	2,932,592	38.0	187,202	105	1,073,475	NextSeq 500	FastQC v0.11.5, bbduk v38.89	DAKLUI000000000	SAMN31902386	GCA_026550015.1	SRR22438356
OLM102	2	2	4b	1955	Clinical, infant, Nova Scotia, CA	+	−	+	65	3,021,434	37.9	96,647	95	984,020	NextSeq 500	FastQC v0.11.5, bbduk v38.89	DAKLVX000000000	SAMN31902336	GCA_026550835.1	SRR22438316
OLM117	2	2	4b	1956	Clinical, infant, Nova Scotia, CA	+	−	+	36	2,992,709	37.9	177,062	97	997,636	NextSeq 500	FastQC v0.11.5, bbduk v38.89	DAKLWS000000000	SAMN31902339	GCA_026551255.1	SRR22438293
OLM118	2	2	4b	1956	Human clinical, Nova Scotia, CA	+	−	+	52	2,990,616	37.9	137,056	103	1,055,572	NextSeq 500	FastQC v0.11.5, bbduk v38.89	DAKLWU000000000	SAMN31902340	GCA_026551275.1	SRR22438292
OLM120	2	2	4b	1957	Clinical, infant, Ontario, CA	+	−	+	141	3,050,343	37.8	41,625	120	1,261,946	NextSeq 500	FastQC v0.11.5, bbduk v38.89	DAKLWT000000000	SAMN31902341	GCA_026551295.1	SRR22438291
OLM121	2	2	4b	1957	Human clinical, Ontario, CA	+	−	+	100	3,063,616	37.8	61,834	129	1,378,821	NextSeq 500	FastQC v0.11.5, bbduk v38.89	DAKLWV000000000	SAMN31902342	GCA_026551315.1	SRR22438290
OLM124	2	2	4b	1958	Clinical, infant, Ontario, CA	+	−	+	66	2,992,623	37.9	82,395	66	904,696	NextSeq 500	FastQC v0.11.5, bbduk v38.89	DAKLWX000000000	SAMN31902343	GCA_026551355.1	SRR22438289
OLM127	2	2	4b	1959	Hare, Newfoundland, CA	+	−	+	67	2,967,175	37.9	102,410	91	925,822	NextSeq 500	FastQC v0.11.5, bbduk v38.89	DAKLVV000000000	SAMN31902346	GCA_026550815.1	SRR22438314
OLM138	2	2	4b	1961	Clinical, infant, Ontario, CA	+	−	+	106	2,954,382	37.9	56,984	80	807,042	NextSeq 500	FastQC v0.11.5, bbduk v38.89	DAKLWG000000000	SAMN31902351	GCA_026550955.1	SRR22438309
OLM151	2	2	4b	1963	Clinical, infant, Newfoundland, CA	+	−	+	97	3,033,300	37.8	63,987	92	954,916	NextSeq 500	FastQC v0.11.5, bbduk v38.89	DAKLWJ000000000	SAMN31902357	GCA_026551075.1	SRR22438302
OLM153	2	2	4b	1963	Human clinical, Newfoundland, CA	+	−	+	79	3,051,861	37.8	69,915	102	1,064,177	NextSeq 500	FastQC v0.11.5, bbduk v38.89	DAKLWL000000000	SAMN31902359	GCA_026551115.1	SRR22438300
OLM157	2	2	4b	1964	Clinical, Newfoundland, CA	+	−	+	61	3,003,307	37.9	83,818	103	1,065,005	NextSeq 500	FastQC v0.11.5, bbduk v38.89	DAKLWK000000000	SAMN31902360	GCA_026551135.1	SRR22438299
OLM77	2	2	4b	1954	Human clinical, Ontario, CA	+	−	+	40	3,007,989	37.9	206,912	102	1,052,631	NextSeq 500	FastQC v0.11.5, bbduk v38.89	DAKLUN000000000	SAMN31902396	GCA_026550035.1	SRR22438354
OLM78	2	2	4b	1954	Clinical, Ontario, CA	+	−	+	55	3,006,421	37.9	136,667	84	864,547	NextSeq 500	FastQC v0.11.5, bbduk v38.89	DAKLUJ000000000	SAMN31902397	GCA_026550055.1	SRR22438353
OLM85	2	2	4b	1953	Human clinical, TX, USA	ND	ND	ND	21	2,976,391	37.9	448,286	82	518,374	Illumina MiSeq	BioNumerics 7.6.3	ABJHYO000000000	SAMN31669759	GCA_026109265.1	SRR22245247
OLM144	**2998**	2	4b	1961	Human clinical, Brazil	+	+	+	51	2,966,194	37.9	91,356	105	1,065,183	NextSeq 500	FastQC v0.11.5, bbduk v38.89	DAKLWH000000000	SAMN31902355	GCA_026551035.1	SRR22438305
OLM36	4	4	4b	1940	Human clinical, Argentina	−	−	−	126	2,896,778	38.0	43,552	56	558,366	NextSeq 500	FastQC v0.11.5, bbduk v38.89	DAKLVK000000000	SAMN31902368	GCA_026550555.1	SRR22438324
OLM44	4	4	4b	1950	Ferret, USA	−	−	−	97	2,914,945	38.0	56,467	107	1,080,192	NextSeq 500	FastQC v0.11.5, bbduk v38.89	DAKLVU000000000	SAMN31902375	GCA_026550775.1	SRR22438317
OLM12	118	4	4b	1934	Human clinical, CT, USA	+	−	+	58	2,943,210	37.9	100,839	80	799,304	NextSeq 500	FastQC v0.11.5, bbduk v38.89	DAKLVT000000000	SAMN31902406	GCA_026550635.1	SRR22438367
OLM30	**2967**	4	4b	1938	Human clinical, Uruguay	−	−	−	115	2,886,375	37.9	55,002	63	627,334	NextSeq 500	FastQC v0.11.5, bbduk v38.89	DAKLUY000000000	SAMN31902366	GCA_026550335.1	SRR22438336
OLM115	**1258**	1258	4b	1955	Clinical, infant, Nova Scotia, CA	+	−	+	57	2,909,722	37.9	94,066	103	1,025,457	NextSeq 500	FastQC v0.11.5, bbduk v38.89	DAKLVY000000000	SAMN31902337	GCA_026550875.1	SRR22438315
OLM116	**1258**	1258	4b	1956	Clinical, infant, Nova Scotia, CA	+	−	+	76	2,910,428	37.9	88,079	81	809,875	NextSeq 500	FastQC v0.11.5, bbduk v38.89	DAKLWD000000000	SAMN31902338	GCA_026551015.1	SRR22438304
OLM80	**1258**	1258	4b	1954	Human clinical, Ontario, CA	+	−	−	100	2,908,797	37.9	76,743	122	1,229,177	NextSeq 500	FastQC v0.11.5, bbduk v38.89	DAKLUK000000000	SAMN31902398	GCA_026550075.1	SRR22438352
OLM82	201	69	Lineage III	1953	Jena, Germany	ND	ND	ND	86	2,959,147	38.1	67,343	126	1,300,974	NextSeq 500	FastQC v0.11.5, bbduk v38.89	DAKLUL000000000	SAMN31902400	GCA_026550115.1	SRR22438350
OLM22	202	69	Lineage III	1937	Sheep, New Zealand	ND	ND	ND	55	2,985,660	38.0	127,755	138	1,471,915	NextSeq 500	FastQC v0.11.5, bbduk v38.89	DAKLWM000000000	SAMN31902361	GCA_026551155.1	SRR22438298
OLM21	**2949**	69	Lineage III	1937	Sheep, USA	ND	ND	ND	30	2,974,255	38.1	224,758	88	887,373	NextSeq 500	FastQC v0.11.5, bbduk v38.89	DAKLUB000000000	SAMN31902411	GCA_026549895.1	SRR22438361
OLM84	144	131	Lineage III	1949	Human clinical, MD, USA	ND	ND	ND	79	2,898,366	38.2	69,888	112	1,120,493	NextSeq 500	FastQC v0.11.5, bbduk v38.89	DAKLUO000000000	SAMN31902401	GCA_026550135.1	SRR22438349
OLM5	623	131	Lineage III	1933	Fowl, USA	−	−	−	61	2,867,574	38.1	84,376	115	1,144,987	NextSeq 500	FastQC v0.11.5, bbduk v38.89	DAKLUC000000000	SAMN31902413	GCA_026549955.1	SRR22438359
OLM6	623	131	Lineage III	1933	Fowl, USA	−	−	−	23	2,881,443	38.1	231,165	204	1,983,871	NextSeq 500	FastQC v0.11.5, bbduk v38.89	DAKLUG000000000	SAMN31902414	GCA_026549975.1	SRR22438358
OLM39	**2968**	157	Lineage III	1939	Pig, Iowa, USA	−	−	−	37	2,857,793	38.0	185,227	113	1,146,990	NextSeq 500	FastQC v0.11.5, bbduk v38.89	DAKNCU000000000	SAMN31902371	GCA_026571555.1	SRR22438321
OLM37	1263	1263	Lineage III	1937	Sheep, Iowa, USA	−	−	−	53	2,904,612	37.9	120,801	88	883,508	NextSeq 500	FastQC v0.11.5, bbduk v38.89	DAKLVJ000000000	SAMN31902369	GCA_026550575.1	SRR22438323
OLM8	**2948**	2824	Lineage III	1933	Sheep, USA	−	−	−	32	2,889,416	38.0	152,612	90	887,229	NextSeq 500	FastQC v0.11.5, bbduk v38.89	DAKLUH000000000	SAMN31902415	GCA_026549995.1	SRR22438357
OLM62	**2971**	2971	Lineage III	1953	Cow, Ontario, CA	ND	ND	ND	44	2,895,568	38.0	121,958	90	893,938	NextSeq 500	FastQC v0.11.5, bbduk v38.89	DAKLVH000000000	SAMN31902384	GCA_026550515.1	SRR22438326
OLM59	**2970**	2970	Lineage IV	1953	Human clinical, Ontario, CA	ND	ND	ND	83	2,782,278	37.9	65,052	113	1,072,602	NextSeq 500	FastQC v0.11.5, bbduk v38.89	DAKLVF000000000	SAMN31902382	GCA_026550475.1	SRR22438328

^*a*^ Sequence types (STs) highlighted in bold are novel STs first identified during this project.

^*b*^ CC, clonal complex.

^*c*^ Serotype designations were determined by PCR (15) and confirmed via core genome multilocus sequence typing (cgMLST)-based clustering analysis using BIGSdb-Lm, hosted by Institut Pasteur (https://bigsdb.pasteur.fr/listeria/) (13).

^*d*^ Strains were tested for resistance against cadmium [35 and 70 µg/mlL – (Cd35/70)], arsenic [500 µg/mlL – (As500)], and a quaternary ammonium compound [10 µg/mlL benzalkonium chloride – (BC10)]. “+” indicates resistance while “−“ indicates sensitivity to the corresponding compound at the specified concentration. ND, not determined.

## Data Availability

This Whole Genome Shotgun project has been deposited in GenBank under the accession numbers found in Table 1. The versions described in this paper are the first version.
